# Chylothorax associated with lymphatic reflux in a thoracic duct tributary after lung cancer surgery

**DOI:** 10.1111/1759-7714.14062

**Published:** 2021-06-21

**Authors:** Hironori Ishida, Ken Nakazawa, Akitoshi Yanagihara, Tetsuya Umesaki, Ryo Taguchi, Akiko Yamada, Hiroyuki Nitanda, Hirozo Sakaguchi

**Affiliations:** ^1^ Department of General Thoracic Surgery Saitama Medical University International Medical Center Saitama Japan; ^2^ Department of Diagnostic Radiology Saitama Medical University International Medical Center Saitama Japan

**Keywords:** chylothorax, lung, lymphatic vessels, lymphography, thoracic duct

## Abstract

Chyle leaks are attributed to damage to the thoracic duct itself or its tributaries during surgery. Chylothorax after lung cancer surgery can occur due to damaged thoracic duct tributaries; however, little is known of the mechanism involved. A 71‐year‐old female underwent a left upper lobectomy with hilar and mediastinal lymphadenectomy for a 1.8‐cm primary squamous cell carcinoma, and developed a chylothorax a day later. Catheter lymphangiography revealed high‐flow chyle leaks from a damaged thoracic duct tributary, known as a bronchomediastinal lymph trunk, due to a lymphatic reflex from the thoracic duct. Subsequently, catheter embolization of the tributary repaired the chylothorax. The potential for persistent chylothorax after lung cancer surgery and successful lymphatic intervention should be noted.

## Graphical abstract

Chylothorax after lung cancer surgery can be associated with lymphatic reflux into the damaged tributary from the thoracic duct. Catheter lymphangiography proved useful in detecting the location and direction of flow of lymphatic leakages as well as that in subsequent embolization.

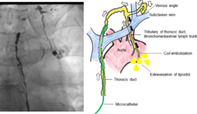

## INTRODUCTION

Chylothorax after pulmonary resection and lymph node dissection for lung cancer is a rare but potentially life‐threating complication.^1^ Although chyle leaks have been attributed to damage to the thoracic duct itself or its tributaries during surgery,^2^ little is known on how the latter is implicated in chylothorax. Recently, lymphangiography and lymphatic intervention have been used in the diagnosis and treatment of postsurgical chyle leaks.^3^ Herein, we report a case of postoperative chylothorax due to chyle leaks from damage to a thoracic duct tributary, known as a bronchomediastinal lymph trunk.^4^ Transcatheter thoracic ductography proved useful for the visualization of chylous lymph reflux from the thoracic duct and for the embolization of the injured tributary.

### CASE REPORT

**FIGURE 1:**
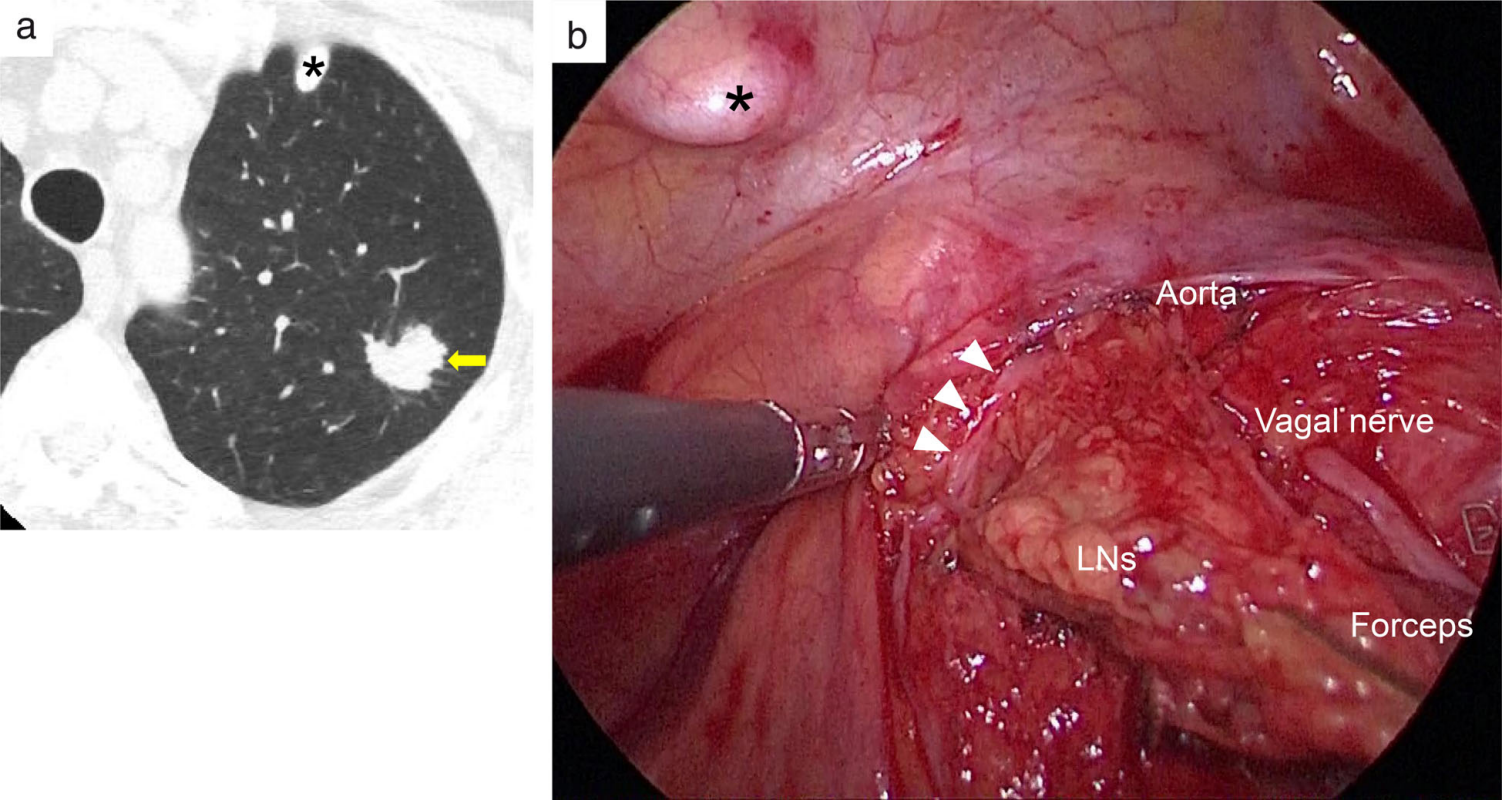
Preoperative computed tomography and intraoperative video images. The asterisk indicates an ossified tip of the first rib. (a) A solid nodule (arrow), 1.8 cm in diameter, in the left upper pulmonary lobe is shown. (b) Postoperative video inspection revealed that moving para‐aortic and subaortic lymph nodes to the back with forceps allowed a glimpse of a lymphatic vessel (arrowheads) running along the ascending aorta. LNs, lymph nodes

**FIGURE 2:**
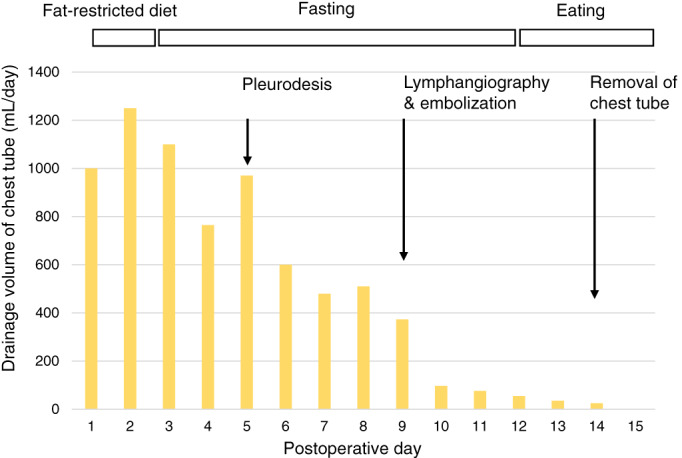
Illustration depicting clinical course. Diet, drainage volume, and treatment for the chylothorax are shown according to the number of days after lung cancer surgery. A considerable reduction in drainage volume by lymphangiography and embolization is noted

A 71‐year‐old asymptomatic female with diabetes mellitus and a 42‐pack‐year smoking history was referred to our hospital for treatment of an abnormal pulmonary nodule measuring 1.8 cm (Figure 1(a)). After a detailed examination, we performed a left upper lobectomy with hilar and mediastinal lymph node dissection (Figure 1(b)) for a primary pulmonary squamous cell carcinoma. The case was subsequently found to be p‐T1bN0M0, stage IA2. After oral intake on postoperative day (POD) 1, a chylous pleural effusion that was drained through a chest tube, increasing to 1000 ml per day, was diagnosed as evidence of a chylothorax (Figure 2). A fat‐restricted diet and fasting followed by pleurodesis with OK‐432 (Picibanil; Chugai Pharmaceutical Co.) between POD 2–8 allowed pleural drainage to decrease to 500 ml per day, but the chylothorax failed to resolve.

**FIGURE 3:**
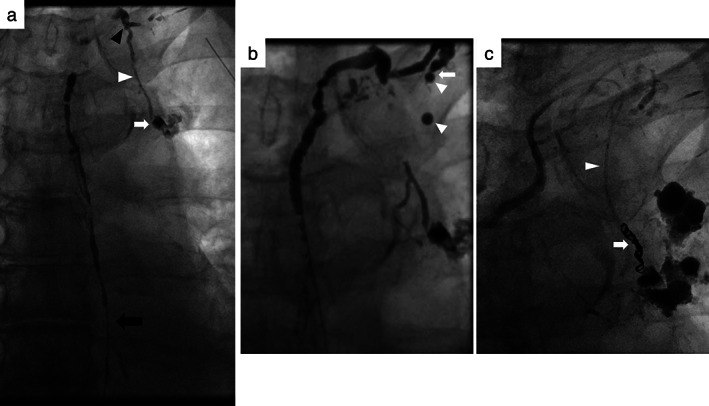
Fluoroscopic images. (a) Thoracic ductography with a Lipiodol injection into the thoracic duct via microcatheter (black arrow) revealed no leakage from the thoracic duct itself, but did show extravasation (white arrow) from the para‐aortic site of its tributary, the bronchomediastinal lymph trunk (white arrowhead), which joins the thoracic duct (black arrowhead). (b) The thoracic duct, which normally terminates at a left venous angle with a beak‐shaped valve (arrow), is shown. Note two droplets of Lipiodol (arrowheads) passing through in the subclavian vein. (c) Microcoils (arrow), used for embolization proximal to the leakage point with a microcatheter (arrowhead) through the tributary, are shown

We therefore conducted lymphangiography and a lymphatic intervention on POD 9 before deciding on surgical repair (Figure 2). A right inguinal lymph node was punctured with a 25 G needle under ultrasound and then 5 ml of Lipiodol (ethiodized oil; Guerbet) was slowly injected under fluoroscopy. After visualization of cisterna chyli and the thoracic duct within 20 min, a needle puncture of the thoracic duct via a percutaneous transabdominal route enabled catheterization for lymphatic intervention. Thoracic ductography using a microcatheter revealed an intact thoracic duct; however, a tributary that leaked contrast media where mediastinal lymph nodes had been dissected during surgery was observed (Figure 3(a), (b)). Such lymph nodes correspond to para‐aortic (no. 6) and subaortic (no. 5) nodes according to a lymph node map in the seventh TNM classification (Figure 1(b)).^5^ Notably, this tributary, termed a bronchomediastinal lymph trunk, was shown to join the thoracic duct prior to its venous termination. Embolization of this tributary was carried out using four microcoils, followed by the injection of N‐butyl cyanoacrylate mixed with Lipiodol (Figure 3(c)). Lymphangiography and embolization procedures each took 40 min.

**FIGURE 4:**
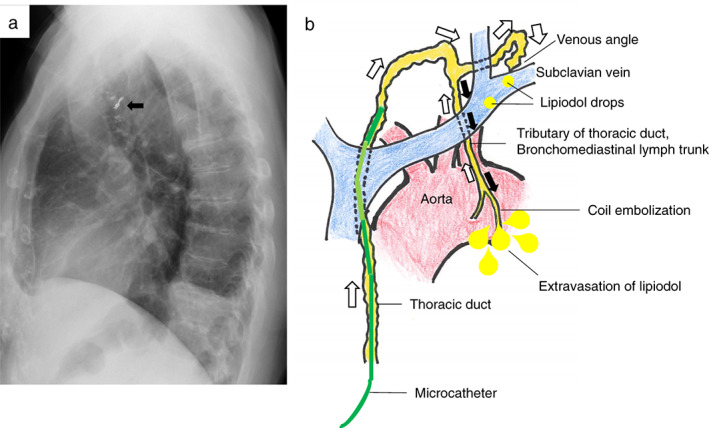
(a) Lateral view of a chest X‐ray a year after embolization shows microcoils (black arrow) on an aortic shadow. (b) Illustration of mechanisms that contributed to the chylothorax. The yellow‐colored vessels and drops represent the contrast medium, Lipiodol, and suggest lymph fluids, namely, chyle. White and black arrows show normal antegrade and causative retrograde chyle flows, respectively

The volume of fluid drained from the chest subsequently decreased to less than 100 ml per day, starting one day after the intervention, eventually leading to the removal of the chest tube five days after embolization (POD14; Figure 2). The patient subsequently had an uneventful 13 months after discharge (Figure 4(a)).

## DISCUSSION

Chylothorax after lung cancer surgery can be associated with chyle–lymph reflux into the injured tributary from the thoracic duct. Lymphangiography followed by lymphatic embolization is useful in the detection and subsequent treatment of this condition.

Anatomically, the thoracic duct commonly terminates at a left venous angle, where valves prevent a retrograde flow of venous blood,^6^ as found in the present case (Figure 3(b)). Given that its tributary lymph vessels are also normally valved, lymphatic fluids in the thoracic duct cannot flow back into the tributaries.^4^, ^7^ This potentially explains why the development of a chylothorax is quite rare after lung cancer surgery. In a study of cadavers, however, Riquet et al.^4^ reported that chylothorax after mediastinal lymphadenectomy can be caused by lymphatic reflux at damaged sites of tributaries that show incompetent lymphatic valves, as found in our case (Figure 4(b)). Some tributaries, however, even lack valves altogether. Indeed, another morphological study uncovered an absence of valvular structures in several subsidiary lymph trunks that drained into the thoracic duct, indicating a possible contribution to retrograde chyle leaks following neck dissections.^6^

Lymphangiography is useful for the diagnosis and treatment of chyle leaks. Lipiodol lymphangiography itself has therapeutic efficacy for various chyle leaks, probably due to Lipiodol acting as an embolic agent.^3^ Recently, however, as an alternative means of accessing the lymphatic system, transcatheter thoracic ductography and embolization have been shown to assist in a high‐output chylothorax due to iatrogenically damaged thoracic duct collaterals.^8^ A high‐flow chylothorax of >500 ml/day, as in our case, generally provides a benchmark for coil embolization at our hospital. Indeed, high‐output extravasation of iodinated contrast medium from the tributary was visualized live under fluoroscopy. Subsequently, microcatheter access to the extravasated, chyle leakage point allowed coil embolization. To our knowledge, this is the first case of chylothorax after lung cancer surgery in which catheter lymphangiography revealed a causative lymphatic reflux, leading to successful embolization.

In our surgery, mediastinal lymph nodes and lymphatic vessels have been usually dissected using an ultrasonic scalpel (energy device) and/or electrocautery. In addition, ligatures have been treated especially for markedly dilated lymphatic vessels. In the present case, however, all lymph nodes had been dissected using only an ultrasonic scalpel. Video review after the development of a chylothorax revealed a somewhat dilated lymphatic vessel, which had been overlooked during surgery, in the para‐aortic (no. 6) and subaortic (no. 5) node area (Figure 1(b)). This lymphatic vessel may indicate an injured thoracic duct tributary that was potentially associated with a chylothorax. Therefore, during surgery whenever an increase is found in the number of lymphatic vessels, dilated or not, around lymph nodes, such lymphatic vessels are occluded by clamping with clips or ligatures. This can prevent lymphatic reflex, given the existence of valve incompetency.

Except for lung surgery, chylothorax after esophagectomy is probably due to direct injury of the thoracic duct, whereas chylothorax after a median sternotomy in cardiac surgeries might be associated with valve incompetency of injured thoracic duct tributaries.^4^

In conclusion, whether persistent chylothorax associated with damage to thoracic duct tributaries occurs on a frequent basis remains to be determined. However, chylothorax after lung cancer surgery can be explained by lymphatic reflux. We found that catheter lymphangiography proved invaluable in detecting the location and flow direction of lymphatic leakages as well as in the treatment of our patient.

## CONFLICT OF INTEREST

The authors report no conflict of interest.
